# Different Alkali Carbonates on the Microstructure and Photoluminescence Properties of SrWO_4_:Tb^3+^ Phosphors

**DOI:** 10.3390/molecules31183214

**Published:** 2026-09-11

**Authors:** Faxue Ma, Xiangju Wu, Jingwei Hu, Ruoyang Li, Dingcheng Yang, Weiwei Shi, Xueqing Zhu, Yongguo Cao, Aiyun Jiang

**Affiliations:** 1 College of Engineering, Huanghe University of Science and Technology, Zhengzhou 454000, China; 2 School of Materials Science and Engineering, Henan University of Science and Technology, Luoyang 471023, China; 3 National Engineering Research Center of Wheat and Corn Further Processing, School of Food Science and Technology, Henan University of Technology, Zhengzhou 450001, China; 4 Henan Zhongfu Industrial Co., Ltd., Gongyi 452100, China

**Keywords:** lanthanides, phosphors, strontium tungsten oxide, luminescence

## Abstract

In this research, terbium-doped strontium tungsten oxide samples were synthesized using the solid-state synthesis method. It was found through X-ray powder diffraction (XRD) that the samples belong to the tetragonal crystal system and have the space group I4_1_/a. The SEM-EDS images indicate that the Na, K, W, and Tb atoms are uniformly distributed throughout the samples. Photoluminescence (PL) measurements were applied for sample characterization. All of them exhibited green emission with the highest peak at 545 nm attributable to the ^5^D_4_ → ^7^F_5_ transition. The highest emission intensity was observed in the Sr_0.996_Tb_0.004_WO_4_ sample, while higher doping levels resulted in a decrease in the PL intensities due to concentration quenching. Samples with additional alkali metal ions for charge compensation were also prepared, and their PL properties were measured; the enhancement of co-doping alkali metal (A^+^ = Li^+^, Na^+^, K^+^) on the green luminescence is demonstrated thoroughly. Doping with 0.5 mol% Na^+^ ions yielded a 9.14-fold increase in peak intensity of 545 nm, a 8.02-fold enhancement in operational lifespan, and a 88.82% improvement in quantum yield. These results indicate that Sr_0.991_Tb_0.004_Na_0.005_WO_4_ materials have potential application as green phosphors in LEDs.

## 1. Introduction

Luminescent materials play a pivotal role in modern optoelectronic devices, particularly in solid-state lighting and display technologies [1,2,3,4]. White light is a blended light of multiple colors and is perceived by human eyes as white light [5]. There are two common approaches to white light generation: (i) blending three monochromatic sources (red, green, and blue), or (ii) utilizing phosphors to convert UV or blue light into a mix of red, green, and blue; or yellow and blue [6,7]. In contrast, single-matrix phosphors can emit blue, green, and red lights that are potential white light sources because they offer greater luminescence efficiency and lower manufacturing costs compared to systems requiring multiple phosphors to accomplish the same effect [8]. Therefore, it is an urgent task to develop a single-phase phosphor that can produce white-light emission [9,10,11].

Lanthanides (Ln^3+^), including Tb, Eu, and Dy, exhibit efficient luminescence due to shielded 4f electrons [12], creating discrete energy levels. Their sharp emissions stem from electric-dipole-forbidden 4f–4f transitions; direct excitation yields low efficiency, while indirect excitation via parity-mixing with the host lattice enhances it [13]. Transition probabilities are sensitive to the local ionic environment [14]. Tb is prominent in optoelectronics for its strong green emission at 543 nm corresponding to ^5^*D*_4_ → ^7^*F*_5_ transitions, alongside peaks at 488, 586, 621, and 650 nm corresponding to ^5^*D*_4_ → ^7^*F*_6_, ^5^*D*_4_ → ^7^*F*_4_, ^5^*D*_4_ → ^7^*F*_3,_ and ^5^*D*_4_ → ^7^*F*_2_ transitions, respectively [13]. Despite these advantages, Tb^3+^-based phosphors commonly exhibit relatively low excitation efficiency and are prone to thermal quenching at elevated operating temperatures, which severely limits their practical applicability [15,16,17].

These limitations arise mainly from the parity-forbidden character of Tb^3+^ 4f-4f transitions and the intense sensitivity of Tb^3+^ emission to the local crystal environment [3]. Local lattice distortion and defect formation frequently initiate non-radiative relaxation pathways, resulting in a suppressed emission intensity and reduced thermal stability. Consequently, extensive research efforts have been dedicated to regulating the local coordination environment of Tb^3+^ to enhance radiative transitions and control non-radiative losses [5,8]. Compositional regulation through co-doping has emerged as an effective strategy for addressing these challenges; for instance, Mn^2+^ co-doped Sr_3_La(PO_4_)_3_ [18], CaAl_2_Si_2_O_8_ [19], Bi/Ln^3+^ co-doped CaY_2_Al_4_SiO_12_ [20], Ca_3_Y(GaO)_3_(BO_3_)_4_ [21], BaY_2_Si_3_O_10_ [22], and Zn^2+^ co-doped Ba_0.75_Sr_0.25_TiO_3_ [23]. In particular, the introduction of alkali-metal ions (Li^+^, Na^+^, and K^+^) provides a simple yet powerful route for tailoring lattice distortion, regulating defect chemistry, and modifying the local symmetry around Tb^3+^ activators [24]. Such regulation has been demonstrated to enhance the emission efficiency and thermal robustness in Tb^3+^-activated systems such as Li^+^-co-doped MgB_4_O_7_ [25], GdPO_4_·H_2_O [26], K^+^-co-doped GdCa_4_O(BO_3_)_3_ [27], Ca_2_LaTaO_6_ [28], A^+^ (A^+^ = Li, Na, K)-co-doped LiSrVO_4_ [24], K_7_SrGd_2_(B_5_O_10_)_3_ [29], CaFCl [30] phosphors, and Li^+^/Na^+^ co-doped K_7_CaGd_2_(B_5_O_10_)_3_ [31], highlighting the importance of controlled lattice engineering in optimizing Tb^3+^ luminescence.

Among various host lattices, tungstate-based compounds have attracted increasing attention due to their excellent chemical stability, thermal robustness, and favorable crystal field environments for rare-earth ion activation [32]. Strontium tungstate (SrWO_4_), with its scheelite structure, provides an ideal framework for efficient energy transfer and minimal non-radiative relaxation [33,34]. Doping with trivalent lanthanide ions such as Tb^3+^ introduces characteristic green emissions arising from the ^5^*D*_4_ → ^7^*F*_4_ transition. Even though several studies have investigated the luminescent properties of Ln^3+^-doped SrWO_4_ [35,36,37,38], to date, no systematic investigation has been reported on the influence of alkali metal doping on the luminescent characteristics of Tb-doped SrWO_4_.

Here, a series of Tb-doped SrWO_4_ phosphors was synthesized by a solid-state reaction method and designated as Sr_1−x−y_Tb_x_A_y_WO_4_ (where x = 0.001, 0.004, 0.007, 0.01, 0.03, 0.05, 0.07, 0.10; A= Li, Na, K; y = 0.005, 0.01, 0.03). The effects of the initial reactant content on the phase compositions, morphologies, and luminescence properties of these samples were investigated. The co-doping of A^+^ by a charge compensator is expected to significantly improve the luminescence properties of Sr_1−x_Tb_x_WO_4_.

## 2. Experimental

Phosphors of general formula Sr_1−x−y_Tb_x_A_y_WO_4_ were synthesized by the solid-state reaction method. Stoichiometric amounts of analytical-grade starting materials (SrCO_3_, WO_3_, Tb_4_O_7_, Li_2_CO_3_, Na_2_CO_3_, K_2_CO_3_) were mixed and ground in an agate mortar. The mixtures were transferred to a crucible and sintered at 950 °C for 8 h. The obtained products were re-ground in an agate mortar and used for further analysis.

Structural properties of Sr_1−x−y_Tb_x_A_y_WO_4_ samples were studied from XRD (BRUKER D8 ADVANCE diffractometer, Bruker AXS GmbH, Karlsruhe, Germany) patterns recorded using CuKα1 radiation (*λ* = 1.5406 Å) in the diffraction limit 10–70° with a scan speed of 0.02°/min in Bragg–Brentano geometry. Surface features of the samples were studied using a field emission scanning electron microscope (Nova Nano SEM-450, FEI Company (now part of Thermo Fisher Scientific), Hillsboro, OR, USA, equipped with XFlash detector 6/10-Bruker Nano GmbH, Berlin, Germany). Compositional analysis was done using EDS measurement (EDS Quantax 200, Germany, Bruker Nano GmbH, Berlin, Germany). The excitation and emission spectra of the samples were measured using an Edinburgh FLS980 (Edinburgh Instruments Ltd., Livingston, UK), spectrofluorometer equipped with a continuous xenon lamp of 450 W for steady state, a pulsed xenon lamp for decay measurements, and a RED photomultiplier tube (Hamamatsu Photonics K.K., Hamamatsu, Japan) to detect the luminescence.

## 3. Result and Discussion

**Figure 1:**
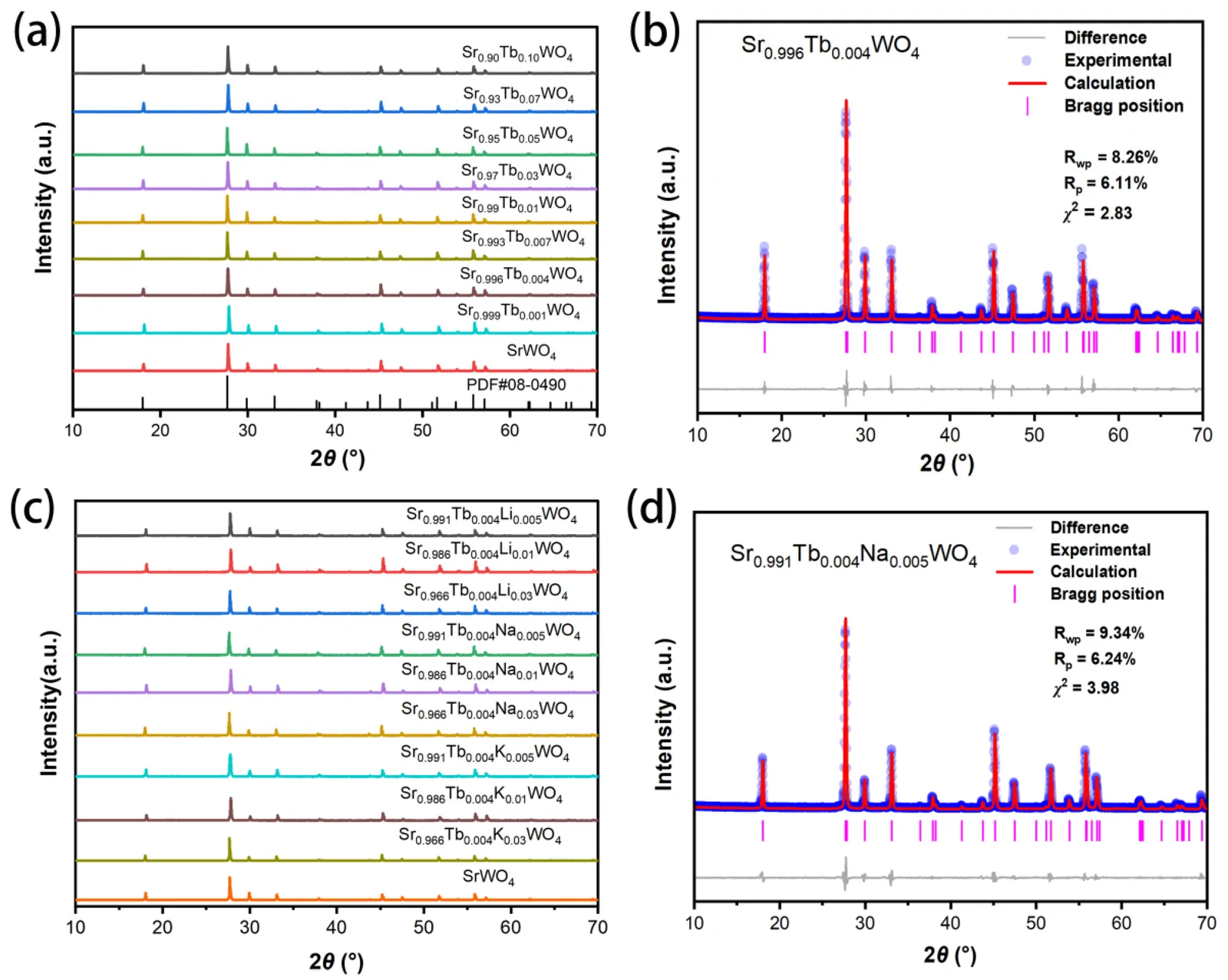
(**a**) XRD pattern of Sr_1−x_Tb_x_WO_4_ (x = 0.001, 0.004, 0.007, 0.01, 0.03, 0.05, 0.07, 0.10) samples; (**b**) the Rietveld refinement fitted XRD of Sr_0.996_Tb_0.004_WO_4_; (**c**) XRD pattern of Sr_0.996-y_Tb_0.004_A_y_WO_4_ (y = 0.005, 0.01, 0.03) samples; (**d**) the Rietveld refinement fitted XRD of Sr_0.991_Tb_0.004_Na_0.005_WO_4_.

XRD is a very powerful characterization technique used to identify the crystal structure of synthesized materials. Figure 1a depicts the XRD patterns of undoped and Tb^3+^-doped SrWO_4_ phosphors. The XRD patterns of all the studied compositions were closely identical, suggesting that all the phosphors under investigation crystallized to a tetragonal crystal structure. The position and relative intensity of all diffraction peaks are in good agreement with the standard values for bulk tetragonal SrWO_4_ (JCPDS 08-0490). No appreciable change in the diffraction patterns of SrWO_4_ with Tb^3+^ ion substitution was observed, indicating that the Tb^3+^ was successfully doped into the host matrix without affecting the parent crystal structure. By considering the ionic radius balance, Tb^3+^ (*r* = 1.04 Å, CN = 8) ions are most likely to replace the Sr^2+^ (*r* = 1.26 Å, CN = 8) site in the host matrix [39,40]. The Rietveld analysis was performed on Sr_0.996_Tb_0.004_WO_4_ as well as Sr_0.991_Tb_0.004_Na_0.005_WO_4_ samples (Figure 1b,d) using the Fullprof package and assuming an I4_1_/a space group for a scheelite-type tetragonal structure. The values of R_wp_, R_p_, and *χ*^2^ are 8.26%, 6.11%, and 2.83 for Sr_0.996_Tb_0.004_WO_4_ and 9.34%, 6.34%, and 3.98 for Sr_0.991_Tb_0.004_Na_0.005_WO_4_, respectively. The Rietveld analysis shows that the samples are in the crystalline phase, and no phase mixture was observed, which confirms the results obtained by conventional XRD. The crystal structure of Sr_0.996_Tb_0.004_WO_4_ is shown in Figure S1. The incorporation of Tb^3+^ and Na^+^ ions induced a marginal lattice expansion, as summarized in Table 1. This table also presents selected crystallographic parameters. As shown in Figure 1c, the PXRD patterns of Sr_0.996_Tb_0.004_WO_4_ samples remained unchanged after doping with Li^+^, Na^+,^ and K^+^, demonstrating no phase transition under those reaction conditions.

**Figure 2:**
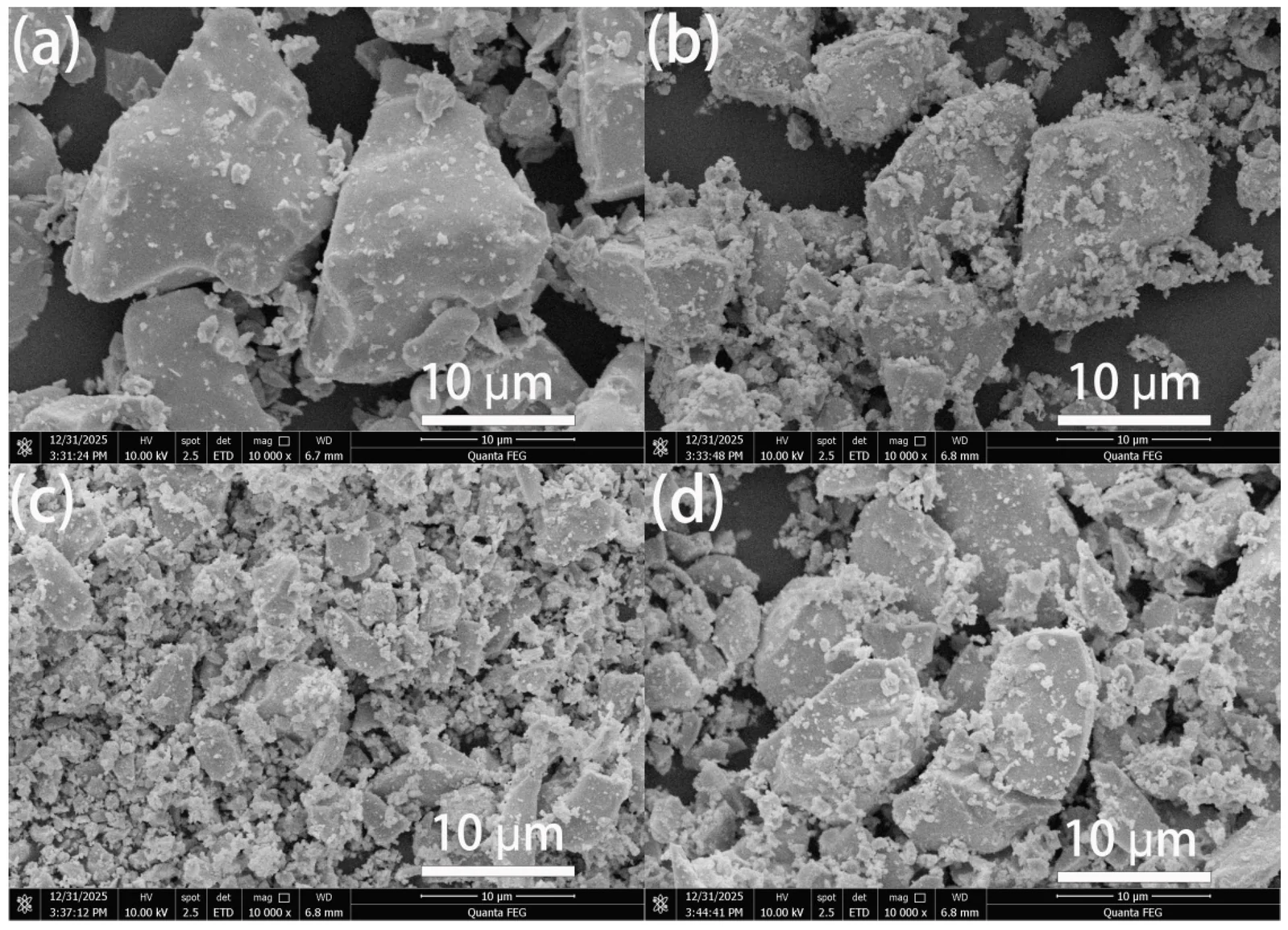
SEM of (**a**) Sr_0.996_Tb_0.004_WO_4_; (**b**) Sr_0.991_Tb_0.004_Li_0.005_WO_4_; (**c**) Sr_0.991_Tb_0.004_Na_0.005_WO_4_; (**d**) Sr_0.991_Tb_0.004_K_0.005_WO_4._

The morphology of Sr_0.996_Tb_0.004_WO_4_ and Sr_0.996-y_Tb_0.004_A_0.005_WO_4_ was studied using SEM. Figure 2 shows that the morphology of particles has no fixed geometry. This observation may be attributed to the mechanical grinding process. The outcome is an inherent consequence of the solid-phase synthesis methodology, wherein bulk solid precursors must be mechanically comminuted into fine powder. Following identical grinding procedures applied to all samples, the influence of alkali metals on particle size was investigated. The size of sample Sr_0.996_Tb_0.004_WO_4_ (Figure 2a) is about 20 × 15 µm, which is significantly larger than the samples doped with alkaline metal elements. With dimensions of 10 × 8 µm and 8 × 5 µm for Sr_0.991_Tb_0.004_Li_0.005_WO_4_ (Figure 2b) and Sr_0.991_Tb_0.004_K_0.005_WO_4_ (Figure 2d), respectively, Sr_0.991_Tb_0.004_Na_0.005_WO_4_ (Figure 2c) possesses a significantly reduced size, 4 × 2 µm, making it the most diminutive component.

**Figure 3:**
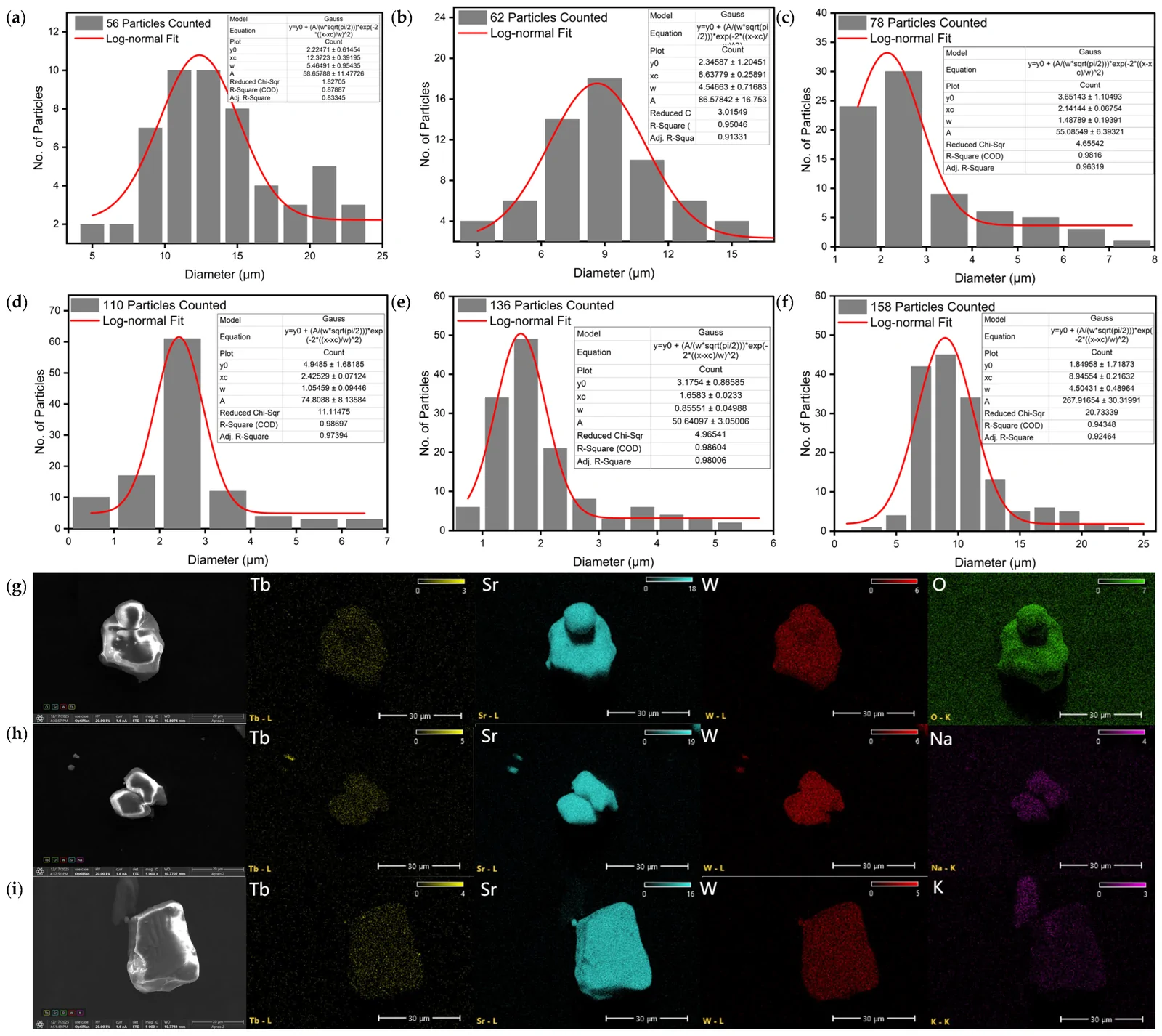
Particle size distribution of (**a**) Sr_0.996_Tb_0.004_WO_4_; (**b**) Sr_0.991_Tb_0.004_Li_0.005_WO_4_; (**c**) Sr_0.991_Tb_0.004_Na_0.005_WO_4_; (**d**) Sr_0.986_Tb_0.004_Na_0.010_WO_4_; (**e**) Sr_0.966_Tb_0.004_Na_0.030_WO_4_; (**f**) Sr_0.991_Tb_0.004_K_0.005_WO_4_; EDS elemental mapping analysis of (**g**) Sr_0.996_Tb_0.004_WO_4_; (**h**) Sr_0.991_Tb_0.004_Na_0.005_WO_4_; (**i**) Sr_0.991_Tb_0.004_K_0.005_WO_4_.

The particle size distribution statistics for these samples are presented in Figure 3a–f. As illustrated in the figure, the particle size of Sr_0.996_Tb_0.004_WO_4_ after grinding is comparable to that of Sr_0.991_Tb_0.004_Li_0.005_WO_4_ and Sr_0.991_Tb_0.004_K_0.005_WO_4_, whereas the particle sizes of Sr_0.991_Tb_0.004_Na_0.005_WO_4_ are markedly reduced. The variation in alkali metal identity is the primary driver of the observed size differences, with its effect overshadowing the more subtle variations induced by changes in Na doping concentration within any given alkali metal system. Moreover, sample Sr_0.996_Tb_0.004_WO_4_ is smoother and has no fine grains adhering to its surface. This phenomenon is attributed to the fluxing action of the alkali metal carbonates [41,42]. Their role as efficient solubilizers enhances precursor dissolution and mass transport, which accelerates the reaction rate and favors the formation of smaller particles. The microscopic size of the prepared phosphor makes it a potential phosphor candidate from the point of view of WLED. The similarity between the sizes derived from XRD and SEM suggests that the particles are well crystallized.

SEM-EDS elemental mapping (Figure 3g–i) showed that both Sr, Tb, Na, and K were uniformly dispersed throughout the corresponding crystals, and no other impurities were observed. This indicates that the Na and K atoms are atomically dispersed within the compound.

**Figure 4:**
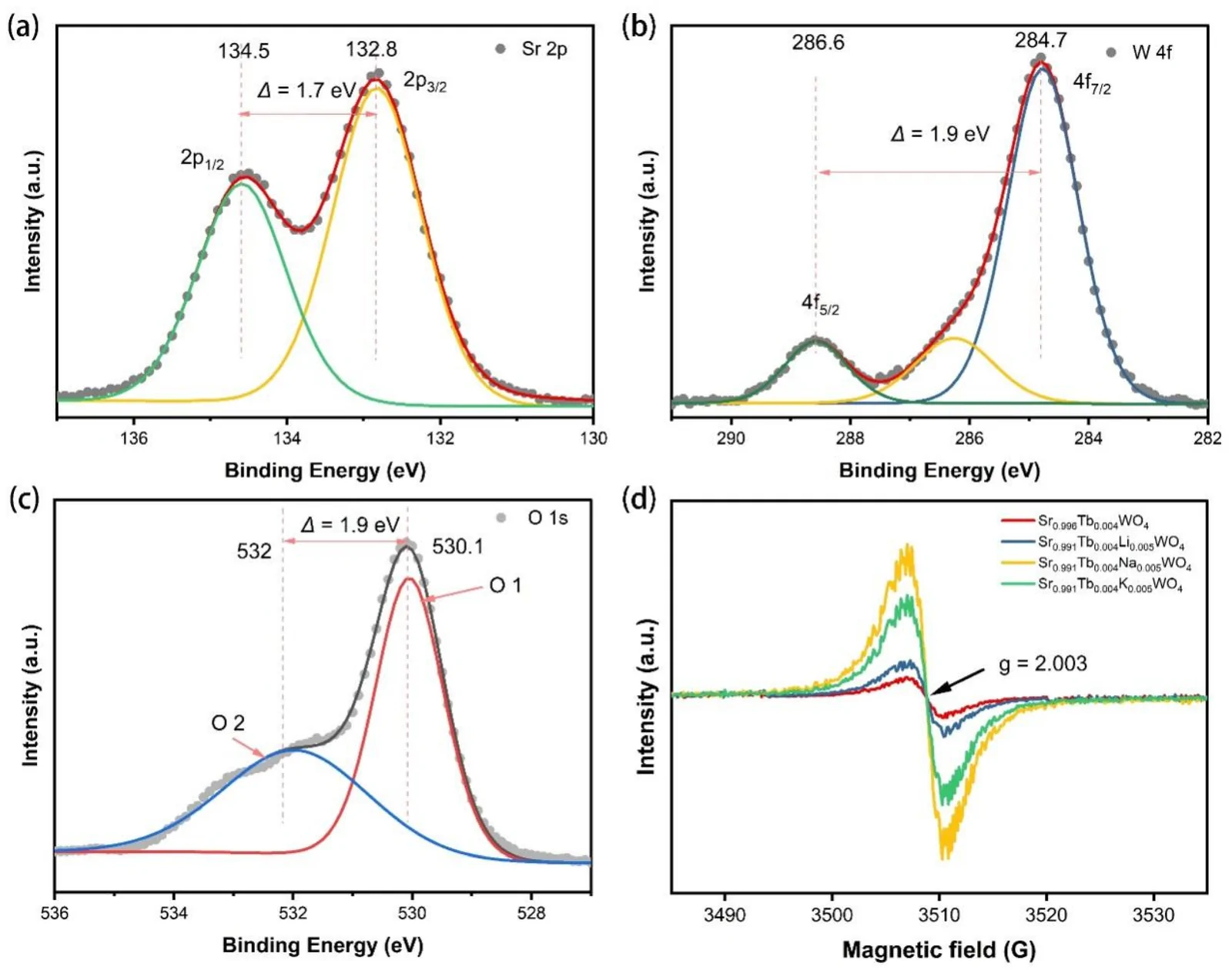
Survey XPS spectrum of Sr_0.996_Tb_0.004_WO_4_, and high-resolution spectra of (**a**) Sr 2p, (**b**) W 4f, (**c**) O 1s, and (**d**) EPR of Sr_0.996_Tb_0.004_WO_4_ and Sr_0.991_A_0.005_Tb_0.004_WO_4_ samples.

XPS analysis of Sr_0.996_Tb_0.004_WO_4_ confirmed the presence and oxidation states of all constituent elements (Figure 4). The high-resolution Sr 2p spectrum (Figure 4a) exhibits peaks at 132.8 and 134.5 eV, assigned to Sr 2p_3/2_ and Sr 2p_1/2_, respectively, with a spin–orbit splitting of 1.7 eV, confirming the +2-oxidation state of Sr. The W 4f peaks (Figure 4b) at 284.7 eV (4f_7/2_) and 286.6 eV (4f_5/2_) with a 1.9 eV splitting indicate W^6+^ in the [WO_4_]^2−^ groups [43]. The O 1s spectrum (Figure 4c) contains two components: a dominant peak at 530.1 eV corresponding to lattice oxygen (O1) and a weaker feature at 532.0 eV associated with defect oxygen (O2) [44]. These results confirm the stable incorporation of Tb^3+^ into the SrWO_4_ lattice, which coexists with [TbO_8_] and [WO_4_] structural units.

The electron paramagnetic resonance (EPR) spectra of the obtained samples present a significant signal at g ≈ 2.003, which is attributed to the paramagnetic singly ionized oxygen vacancy V_O_ (Figure 4d) [45]. The EPR spectra further corroborate the presence of oxygen vacancies, displaying a characteristic signal for the doped samples. Notably, the intensity of this signal is markedly enhanced in the co-doped Sr_0.991_A_0.005_Tb_0.004_WO_4_ compared to the singly doped counterpart, suggesting that the introduction of A ions facilitates the generation of more oxygen vacancies. The introduced defect levels act as effective carrier-trapping sites, which inhibit radiative electron-hole recombination pathways and consequently enhance the emission intensity. Through the synergistic effect of Tb^3+^ doping and A co-doping, the electronic band structure and defect chemistry of the phosphor are jointly optimized, giving rise to outstanding luminescent properties under 369 nm excitation.

**Figure 5:**
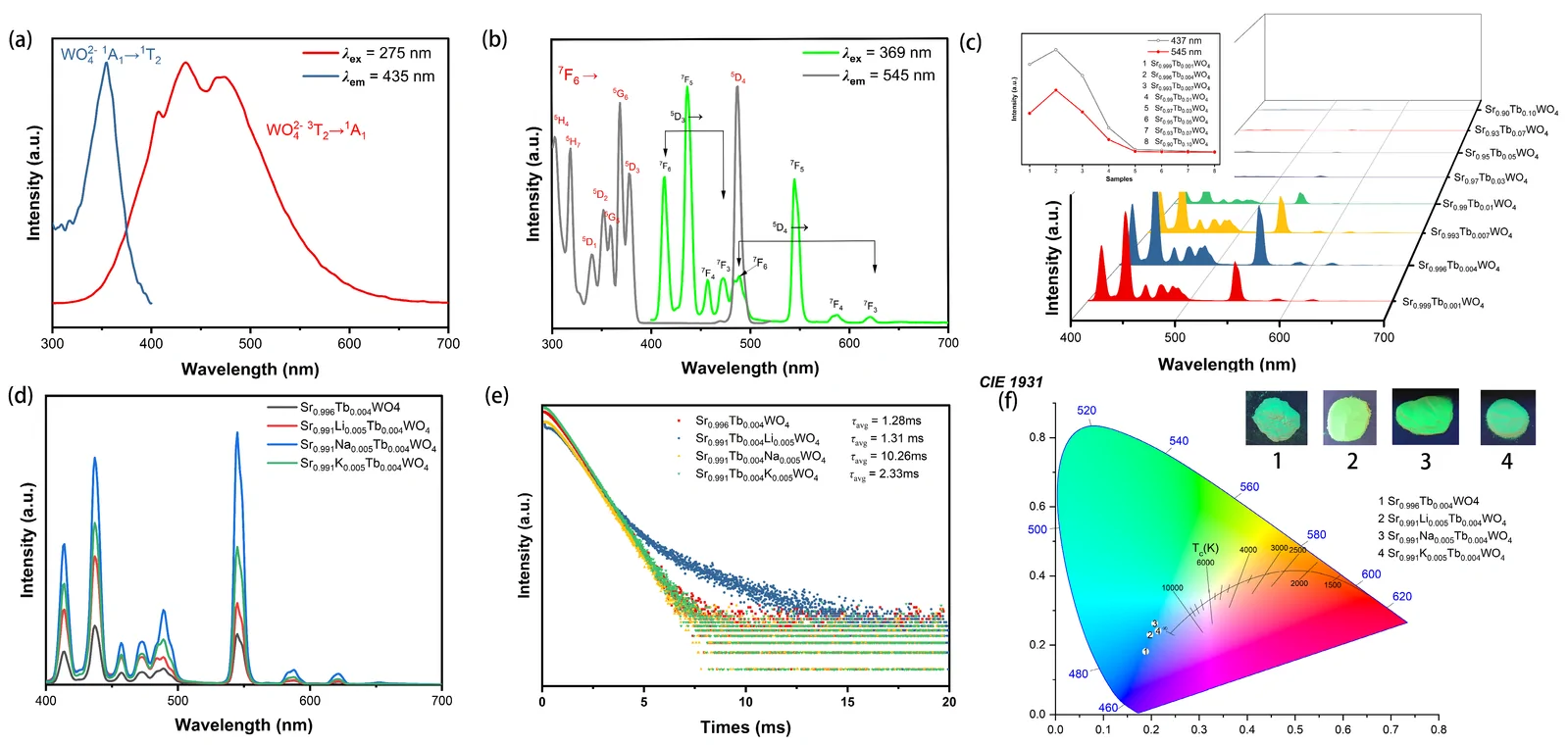
Luminescence spectra of (**a**) SrWO_4_ and (**b**) Sr_0.996_Tb_0.004_WO_4_; (**c**) the emission spectra of SrWO_4_ with different Tb^3+^ concentrations excited at 369 nm (inset: the intensity of the 437 nm and 545 nm peak with different Tb^3+^ concentrations); (**d**) the emission spectra of SrWO_4_ with different alkali metal excited at 369 nm; (**e**) Photoluminescence decay curves for the ^5^D_4_ level (545 nm); (**f**) CIE coordinates of the Sr_0.996_Tb_0.004_WO_4_ and Sr_0.991_Tb_0.004_A_0.005_WO_4_ emission (inset: the intensity of the 437 nm and 545 nm peak with different Tb^3+^ concentrations).

To investigate the luminescence properties of Tb^3+^ doped SrWO_4_ phosphor, a series of samples were prepared. Figure 5a is the excitation spectrum of the SrWO_4_ sample at room temperature with the monitoring wavelength at 435 nm, and the scanning range is 300–400 nm. The excitation band of WO_4_^2−^ is observed at 320–390 nm, resulting from the ^1^A_1_ → ^1^T_2_ energy transition [46], which coincides with the absorption spectrum due to the internal electronic transition in WO_4_^2−^. The emission spectrum of the SrWO_4_ phosphor was excited at 275 nm. The scanning area is 300–700 nm. As shown in the figure, four characteristic peaks located at 411 nm, 438 nm, 468 nm, and 477 nm can be observed within the broad absorption band spanning 330–600 nm, which are attributed to the ^3^T_2_ → ^1^A_1_ electron transition of the WO_4_^2−^ ion [46].

In Figure 5b, the excitation spectrum of Sr_0.996_Tb_0.004_WO_4_ phosphor monitored at 545 nm has a scanning range of 300–520 nm. The presence of the strong band at 303 nm of the WO_4_^2−^ group in the excitation spectrum of Tb^3+^ ions means that there is an energy transfer from the O^2−^ to W^6+^ and from O^2−^ to Tb^3+^. Here, the energy transfer from WO_4_^2−^ to Tb^3+^ is highly efficient and proceeds via three primary mechanisms [47]. The distinct emission spectrum arises from a sequential three-step process: initially, WO_4_^2−^ absorbs ultraviolet light; subsequently, the absorbed energy is transferred to Tb^3+^ ions; and finally, this energy induces the de-excitation of Tb^3+^ ions. Furthermore, a strong and broad excitation band in the ultraviolet region, combined with the characteristic emission of Tb^3+^, enhances the effective excitation of terbium through the sensitizer.

In the region of 300–500 nm, there are some peaks ascribed to the f–f transitions of Tb^3+^, which are assigned to the electron transition from the ^7^F_6_ ground state to the different excitation states as 303 nm ^5^H_4_, 319 nm ^5^H_7_, 340 nm ^5^G_2_, 352 nm ^5^D_2_, 360 nm ^5^G_5_, 369 nm ^5^G_6_, 379 nm ^5^D_3_ and 488 nm ^5^D_4_ [48]. According to the results obtained from the excitation spectrum, 369 nm is chosen as the host sensitization wavelength for the evaluation of the photoluminescence emission characteristics of the Sr_0.996_Tb_0.004_WO_4_ samples. Figure 5b depicts the emission spectra, which consist of four well-defined emission peaks at 414 nm (^5^D_3_ → ^7^F_6_), 437 nm (^5^D_3_ → ^7^F_5_), 457 nm (^5^D_3_ → ^7^F_4_), 473 nm (^5^D_3_ → ^7^F_3_), 489 nm (^5^D_4_ → ^7^F_6_) [40], 545 nm (^5^D_4_ → ^7^F_5_), 587 nm (^5^D_4_ → ^7^F_4_), and 621 nm (^5^D_4_ → ^7^F_3_) due to the distinct intra-configurational ^4^f_8_–^4^f_8_ transitions of Tb^3+^ ions in the host [13,48], respectively. Among these peaks, the green emission corresponds to the ^5^D_4_ → ^7^F_5_ induced electric dipole transition. Hence, the synthesized Tb^3+^ doped SrWO_4_ samples can be used as a potential candidate for WLED application as a green source [49].

Figure S3 presents the Dieke diagram illustrating the energy transfer pathway from the host lattice to Tb^3+^ ions. Upon excitation at 365 nm, electrons initially residing in the O 2p valence band are promoted to the 5d states of W atoms situated near the conduction band minimum. Subsequently, this energy is transferred non-radiatively to higher-lying excited states of Tb^3+^ ions incorporated in the host lattice. Following rapid internal relaxation, population accumulates in the ^5^D_4_ level, resulting in intense green photoluminescence emission characteristic of Tb^3+^ ions [50].

From the emission spectra (Figure 5c), it is evident that the emission intensity increases as the Tb^3+^ concentration rises from 0.1% to 0.4%, then decreases from 0.7% up to 10% doping. This decline in intensity is attributed to concentration quenching [51], which becomes significant at higher doping levels. Consequently, PL excitation and emission studies indicate that 0.4% represents the optimal doping concentration for the Sr_0.996_Tb_0.004_WO_4_ phosphor.

The critical energy transfer distance, i.e., *Rc* is considered by Blasse’s equation given below:

(1) $$Rc\approx 2{\left(\frac{3V}{4\pi \mathrm{XcN}}\right)}^{\frac{1}{3}}$$

where *V*, *N*, and *Xc* are represented as volume, the number of cations that exist in the unit cell, and the critical concentration of quenching of the material, and their values are taken as 350.66 Å^3^, 8, and 0.004, correspondingly. Thus, the critical energy transfer distance is considered as 27.56 Å, much larger than 5 Å, indicating that the interaction between the activator ions can be eliminated. Therefore, the quenching mechanism in theSr_0.996_Tb_0.004_WO_4_ is dominated by the *d-d* interaction.

When trivalent Tb^3+^ ions replace divalent Sr^2+^ ions in the main lattice, it leads to a charge imbalance. The defects or trap states caused by charge imbalance suppress luminescence efficiency, thereby reducing luminescence intensity [52,53]. To address this issue, we introduced alkali metal ions (Li^+^, Na^+,^ and K^+^) as charge compensators. By co-doping alkali metal ions, we effectively neutralized the charge imbalance, thereby stabilizing the crystal structure and changing luminescence intensity. There are no differences in the shape and location of the peaks in the emission spectra between Sr_0.996_Tb_0.004_WO_4_ and Sr_0.996-y_Tb_0.004_A_0.005_WO_4_. All the A^+^ co-doped phosphors displayed identical emission profiles dominated by the hypersensitive ^5^D_4_ → ^7^F_5_ transition at 545 nm, confirming that alkali-ion incorporation does not perturb the crystal-field environment of Tb^3+^. Relative to unmodified Sr_0.996_A_0.005_Tb_0.004_WO_4_, the green emission intensity increased to 161%, 398%, and 172% for Li^+^, Na^+^, and K^+^, following the sequence I_Na_ > I_K_ > I_Li_ (Figure 5d). However, the emission intensity varies significantly depending on the dopant type. As illustrated in Figure S2, for a given alkali metal element, the photoluminescence intensity at 545 nm exhibits a systematic dependence on the doping concentration. Therefore, the emission intensity is dependent on both the identity of the alkali metal and the molar ratio of the dopants [28].

This enhancement arises from the more effective defect neutralization and lattice stabilization achieved when the ionic radius of the compensator closely matches that of Sr^2+^ (1.08 Å); Na^+^ (1.02 Å) provides the best radius compatibility, reducing local strain and maximizing charge-balance efficiency, whereas Li^+^ (0.76 Å) and K^+^ (1.38 Å) introduce slight compressive or tensile distortions that partially limit the effectiveness of compensation.

Enhanced luminescence can be attributed to the following mechanisms [28]. Firstly, charge compensation is achieved through the co-doping mechanism described by the substitution Tb^3+^ + Na^+^ → 2Sr^2+^, which directly contributes to an increase in the emission intensity. Secondly, the incorporation of Na^+^ ions helps suppress the formation of vacancy defects that typically result from the substitution of Tb^3+^ for Sr^2+^. The findings from the EPR experiment further corroborate this conclusion. Thirdly, doping with Na^+^ ions induces a marginal lattice expansion, as evidenced by an increased unit cell volume and interionic spacing (Table 1). This structural effect reduces non-radiative relaxation pathways for the Tb^3+^ ions, thereby further enhancing the overall luminous efficiency.

The impact of A^+^ co-doping on quantum efficiency (QE) corroborates this trend (Table 1). All QE measurements were performed under identical excitation and sample preparation conditions for all the compositions to ensure a reliable comparison. The QE improved from 28.28% for Sr_0.996_Tb_0.004_WO_4_ to 31.76%, 53.40%, and 34.90% for Li^+^-, Na^+^-, and K^+^-compensated samples, respectively, mirroring the intensity enhancement trend. These results confirm that the A^+^ co-doped lattices exhibit fewer non-radiative centers and more efficient energy utilization. The Na^+^-co-doped phosphor demonstrated an optimal balance between electrostatic neutrality and structural coherence, achieving the highest QE and luminescence efficiency in the series.

Fluorescence lifetime analysis (Figure 5e, Table 2) further proves the mechanism of defect neutralization mechanism. The luminescent decay curve of Sr_0.996_Tb_0.004_WO_4_ and Sr_0.991_Tb_0.004_A_0.005_WO_4_ (A = Li, Na, K) can be fitted into a double exponential function *I* = I_0_ +A_1_exp (-t/τ_1_) + A_2_exp (-t/τ_2_). Where I(t) denotes the luminescence intensity at t = t, I_0_ is the initial intensity at t = 0, and t_1_ and t_2_ are the fast- and slow-decay components, respectively, and parameters A_1_ and A_2_ are fitting constants. In the calculation, the τ_avg_ ranged from 1.28 ms for Sr_0.996_Tb_0.004_WO_4_ to 1.31 ms for Li^+^, 10.26 ms for Na^+^, and 2.33 ms for K^+^, showing reduced non-radiative decay with A^+^ ion codoping. The change trend of the average lifetime is *τ*_Na_ > *τ*_K_ > *τ*_Li_, which is consistent with the emission intensity of the phosphor. This may be because Na^+^ can reduce the non-radiative transition of Tb^3+^ ions, while the special doping position of Li^+^ and K^+^ gradually increases the possibility of non-radiative transition. As summarized in Table 2, Na^+^ doping at 0.5 mol% induces a 4.4-fold enhancement in luminescence lifetime and an 88.82% increase in photoluminescence quantum yield. The color changes were further annotated using the Commission Internationale de l’Éclairage (CIE) coordinates as shown in Figure 5f, as the position shifted from (0.19, 0.18) of Sr_0.996_Tb_0.004_WO_4_ to (0.20, 0.23) of Sr_0.991_Tb_0.004_Li_0.005_WO_4_ (0.21, 0.26) of Sr_0.991_Tb_0.004_Na_0.005_WO_4_ and then to (0.21, 0.24) of Sr_0.991_Tb_0.004_K_0.005_WO_4_. Prolonged lifetimes, improved QE, and emission intensity prove that A^+^-ion co-doping decreases the quenching-center density and promotes radiative recombination through the Tb^3+ 5^D_4_ → ^7^F_5_ transition.

## 4. Conclusions

In summary, Sr_1−x−y_Tb_x_A_y_WO_4_ (where x = 0.001, 0.004, 0.007, 0.01, 0.03, 0.05, 0.07, 0.10; y = 0.005, 0.01, 0.03) phosphors were synthesized using the solid-state synthesis method, and their phase purity was confirmed by X-ray diffraction analysis. The particles synthesized by SEM-EDS analysis were about 2–20 μm in size and possessed an irregular morphology. Sr, W, O, and Tb were confirmed in the component analysis. Examination of the luminescent properties of terbium-doped samples showed expected excitation and emission spectra, with the highest emission peak at 544 nm attributable to the ^5^D_4_ → ^7^F_5_ transition. Comparison of emission intensities revealed that the most intense luminescence was observed in the Sr_0.996_Tb_0.004_WO_4_ sample. Charge compensation is achieved through the introduction of Li^+^, Na^+^, or K^+^, which generally results in a slight reduction in emission intensity. Doping with 0.5 mol% Na^+^ ions yielded a 9.14-fold increase in peak intensity at 545 nm, an 8.02-fold enhancement in operational lifespan, and an 88.82% improvement in quantum yield. The luminescence color of Sr_0.996_Tb_0.004_WO_4_ and Sr_0.991_Tb_0.004_A_0.005_WO_4_ powders is located in the green region of the CIE chromatic diagram, so these experimental results imply a potential application of green-emitting optical devices based on SrWO_4_ phosphors.

## Figures and Tables

**Table 1:** Some crystallographic data of SrWO_4_, Sr_0.996_Tb_0.004_WO_4_, and Sr_0.991_Tb_0.004_Na_0.005_WO_4._

	SrWO_4_	Sr_0.996_Tb_0.004_WO_4_	Sr_0.991_Tb_0.004_Na_0.005_WO_4_
Space-group	I4_1/a_ (88)-tetragonal	I4_1/a_ (88)-tetragonal	I4_1/a_ (88)-tetragonal
a (Å)	5.4129	5.4157	5.4168
b	5.4129	5.4157	5.4168
c (Å)	11.9512 (2)	11.9463	11.951
*α*	90	90	90
*β*	90	90	90
*γ*	90	90	90
V (Å^3^)	350.16	350.38	350.66
Z	4	4	4
2*θ* (°)		10–70	10–70
R_wp_		8.26%	9.35%
R_p_		6.11%	6.24%
χ^2^		2.83	3.98

**Table 2:** CIE, Luminescence lifetimes, and quantum efficiencies of Sr_0.996_Tb_0.004_WO_4_ and Sr_0.991_Tb_0.004_A_0.005_WO_4_ samples.

Samples	CIE	Quantum Efficiency	τ_avg_
Sr_0.996_Tb_0.004_WO_4_	(0.19, 0.18)	28.28%	1.28 ms
Sr_0.991_Tb_0.004_Li_0.005_WO_4_	(0.20, 0.23)	31.76%	1.31 ms
Sr_0.991_Tb_0.004_Na_0.005_WO_4_	(0.21, 0.26)	53.40%	10.26 ms
Sr_0.991_Tb_0.004_K_0.005_WO_4_	(0.21, 0.24)	34.90%	2.33 ms

## Data Availability

The data sets used during the current study are available from the corresponding author on reasonable request.

## Supplementary Materials

The following supporting information can be downloaded at: https://www.mdpi.com/article/10.3390/molecules31183214/s1, Figure S1: Construction of the SrWO_4_:0.4%Tb (Sr/Tb: blue; W: gray; O: green): (a) the WO_4_^2−^; (b) the coordination structure of the Sr/Tb atoms; (c) the coordination structure of the O atoms; (d) the coordination structure of the WO_4_^2−^ groups; (e) the connection mode of two WO_4_^2−^ groups; (f) the coordination mode of the WO_4_^2−^ group with Sr and W atoms; (g) the structure of SrWO_4_:0.4%Tb. Figure S2: The emission spectra of SrWO_4_ with different alkali metal excited at 369 nm. Figure S3: Dieke diagram showing the energy transfer mechanism between the host and Tb^3+^ ions. Table S1: ICP data of SrWO_4_, Sr_0.996_Tb_0.004_WO_4_ and Sr_0.991_Tb_0.004_A_0.005_WO_4_ (A = Li^+^, Na^+^, K^+^).
