# Supplementary material for: Different Alkali Carbonates on the Microstructure and Photoluminescence Properties of SrWO4:Tb3+ Phosphors
Source: Molecules. 2026 Sep 11;31(18):3214. doi: 10.3390/molecules31183214 (PMC13609587; doi:10.3390/molecules31183214)
Supplement: Supplementary file 1 [file molecules-31-03214-s001.zip › molecules-4550621-supplementary.pdf]

Different Alkali Carbonates on the Microstructure and Photoluminescence Properties of $SrWO_4:Tb^{3+}$ Phosphors

Faxue Ma [1,*], Xiangju Wu [2], Jingwei Hu [1], Ruoyang Li [1], Dingcheng Yang [1], Weiwei Shi [1], Xueqing Zhu [3,*], Yongguo Cao [4] and Aiyun Jiang [1]

1 College of Engineering, Huanghe University of Science and Technology, Zhengzhou 454000, China
2 School of Materials Science and Engineering, Henan University of Science and Technology, Luoyang 471023, China
3 National Engineering Research Center of Wheat and Corn Further Processing, School of Food Science and Technology, Henan University of Technology, Zhengzhou 450001, China
4 Henan Zhongfu Industrial Co., Ltd., Gongyi 452100, China; caoyongguo@zfgjlc.com
* Correspondence: mfxxmu@163.com (F.M.); zhuxq0816@haut.edu.cn (X.Z.)

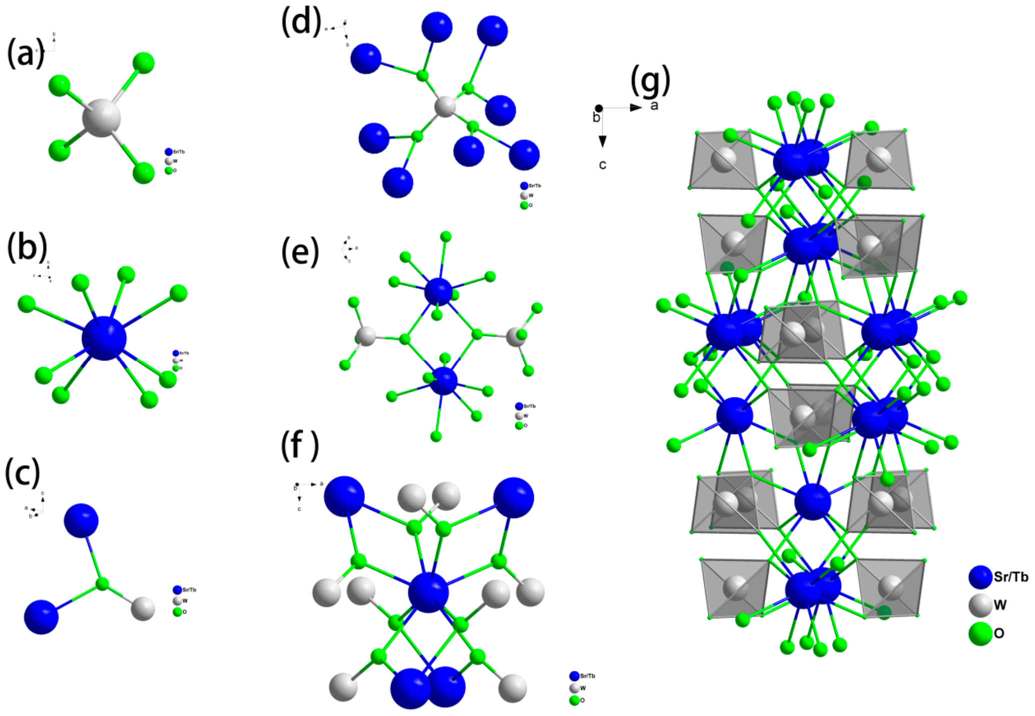

Figure S1. Construction of the $SrWO_4$:0.4%Tb (Sr/Tb: blue; W: gray; O: green.): (a) the WO_4^{2-}; (b) the coordination structure of the Sr/Tb atoms; (c) the coordination structure of the O atoms; (d) the coordination structure of the WO_4^{2-} groups; (e) the connection mode of two WO_4^{2-} groups; (f) the coordination mode of the WO_4^{2-} group with Sr and W atoms; (g) the structure of $SrWO_4$:0.4%Tb.

Table S1. ICP data of $SrWO_4$, $Sr_{0.996}Tb_{0.004}WO_4$ and $Sr_{0.991}Tb_{0.004}A_{0.005}WO_4$ (A = Li^+, Na^+, K^+)

Element	Theoretical Ratio	Testing Ratio
Tb:Sr	4:996	3.76 : 996
Tb:Sr:Li		4.01 : 991 : 4.78
Tb:Sr:Na	4:991:5	3.51 : 991 : 4.89
Tb:Sr:K		4.04 : 991 : 4.40

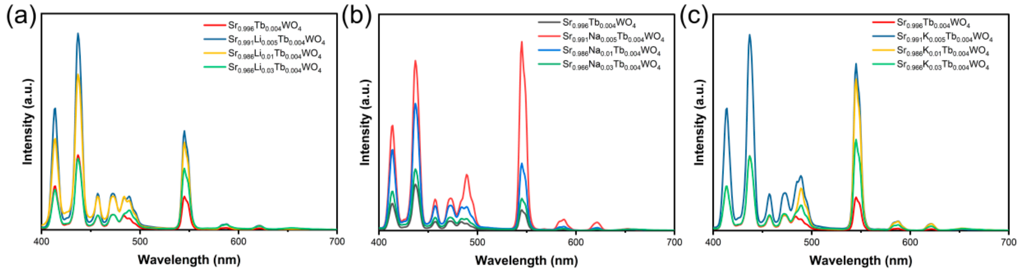

Figure S2. The emission spectra of $SrWO_4$ with different alkali metal excited at 369 nm.

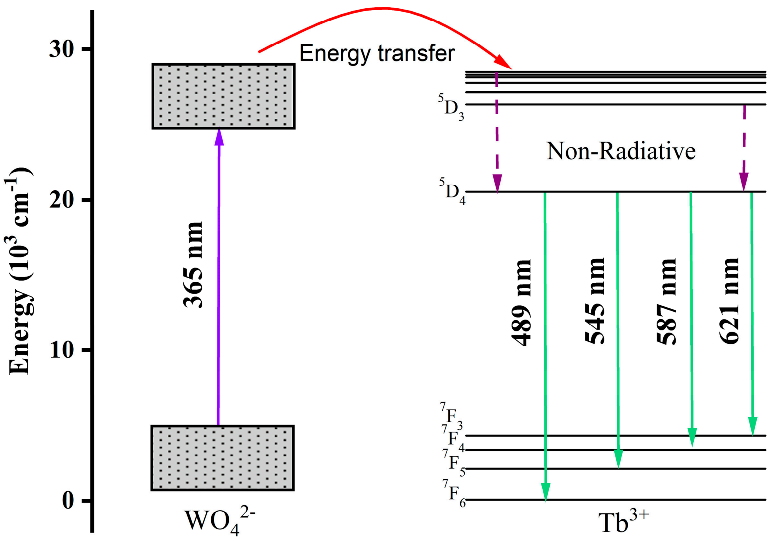

Figure S3. Dieke diagram showing the energy transfer mechanism between the host and Tb^{3+} ions.
